# Comparison of Current International Guidelines on Premature Ejaculation: 2024 Update

**DOI:** 10.3390/diagnostics14161819

**Published:** 2024-08-21

**Authors:** Lorenzo Romano, Davide Arcaniolo, Lorenzo Spirito, Carmelo Quattrone, Francesco Bottone, Savio Domenico Pandolfo, Biagio Barone, Luigi Napolitano, Francesco Ditonno, Antonio Franco, Felice Crocetto, Javier Romero-Otero, Riccardo Autorino, Marco De Sio, Celeste Manfredi

**Affiliations:** 1 Unit of Urology, Department of Woman Child and General and Specialized Surgery, University of Campania “Luigi Vanvitelli”, 80131 Naples, Italy; lorenzo.romano990@gmail.com (L.R.); davide.arcaniolo@gmail.com (D.A.); lorenzospirito@msn.com (L.S.); carmeloquattrone@hotmail.it (C.Q.); bottonefrancesco@yahoo.it (F.B.); marco.desio@unicampania.it (M.D.S.); 2 Department of Urology, University of L’Aquila, 67010 L’Aquila, Italy; pandolfosavio@gmail.com; 3 Division of Urology, Department of Surgical Sciences, AORN Sant’Anna e San Sebastiano, 81100 Caserta, Italy; biagio193@gmail.com; 4 Urology Department, ASL Salerno, 84124 Salerno, Italy; luiginap89@gmail.com; 5 Department of Urology, Azienda Ospedaliera Universitaria Integrata Verona, University of Verona, 37126 Verona, Italy; francesco.ditonno@icloud.com; 6 Department of Urology, Sant’Andrea Hospital, La Sapienza University, 00189 Rome, Italy; 7 Department of Neurosciences, Reproductive Sciences and Odontostomatology, University of Naples “Federico II”, 80131 Naples, Italy; felice.crocetto@unina.it; 8 Department of Urology, HM Hospitales, 28050 Madrid, Spain; jromerootero@hotmail.com; 9 Department of Urology, Rush University Medical Center, Chicago, IL 60612, USA; ricautor@gmail.com

**Keywords:** early, ejaculation, guidelines, IELT, premature, recommendations

## Abstract

Premature ejaculation (PE) is a common male sexual dysfunction that can cause significant distress in the patient and partner. This study aimed to compare the current international guidelines on PE to highlight their similarities and differences. We examined the latest guidelines from the European Association of Urology (EAU), American Urological Association/Sexual Medicine Society of North America (AUA/SMSNA), and International Society of Sexual Medicine (ISSM) by comparing definitions, classifications, epidemiology, pathophysiology, and recommendations on diagnosis and therapy. The quality of guidelines was assessed using the Appraisal of Guidelines for Research and Evaluation (AGREE) Global Rating Scale (GRS). We found significant variations in the definitions of PE and recommendations on management of patients. The EAU guidelines were the most recent, the AUA/SMSNA guidelines lacked detail in some areas, and the ISSM guidelines were the most complete but also the least updated. The search for a unified definition and the development of standardized diagnostic and therapeutic pathways remain concrete issues to improve the management of patients with PE worldwide.

## 1. Introduction

Premature ejaculation (PE) is a male sexual dysfunction whose definition is not universally accepted. However, it is reasonable to affirm that it is characterized by ejaculation that occurs within a relatively short time after the onset of sexual stimulation, inability to delay it, and consequent distress [1]. Likewise, over time, different time cut-offs and classifications have been proposed by different authors [2].

PE is certainly a common sexual dysfunction, but it is difficult to estimate its real prevalence, due to both the equivocal definition and the low percentage of men who seek professional help. Therefore, it remains probably an underdiagnosed condition [3,4]. The risk factors and pathophysiological mechanisms underlying PE are also still unclear [5].

Obviously, PE negatively influences the patient’s sexual health, but severe cases can also prevent ejaculation into the vagina, thereby affecting reproductive function [6]. Overall, it can negatively impact the quality of life (QoL) of both the man and the couple [7]. Therefore, correct diagnosis and appropriate treatment of patients affected by PE appear essential.

There are currently several guidelines from various scientific societies on the topic. These are heterogeneous, indicating diagnostic and therapeutic approaches that are often inconsistent. Investigating the common and discordant points among the available guidelines is essential for identifying where research and expert efforts should focus to obtain shared recommendations and ultimately improve the management of PE.

The aim of this study was to compare a selection of current international guidelines on PE to highlight similarities and differences, with particular attention to the recommendations/statements regarding diagnostic and therapeutic pathways.

## 2. Materials and Methods

We selected the most widely known international guidelines on PE: the European Association of Urology (EAU) guidelines [8], the American Urological Association/Sexual Medicine Society of North America (AUA/SMSNA) guidelines [9], and the International Society of Sexual Medicine (ISSM) guidelines [10]. This selection was agreed upon by the authors after a discussion. The most up-to-date versions of the guidelines at the time of the study (April 2024) were chosen: EAU guidelines 2024, AUA/SMSNA guidelines 2022, and ISSM guidelines 2014. National guidelines, manuals (e.g., the Diagnostic and Statistical Manual of Mental Disorders [DSM] [11], European Society for Sexual Medicine [ESSM] Manual of Sexual Medicine [12]), and disease classifications (e.g., International Classification of Diseases [ICD] [13]) were excluded.

The definition, classification, and the most relevant epidemiological and pathophysiological data of PE reported in each guideline were recorded. Recommendations/statements on the diagnosis and therapy of PE were compared among the guidelines. The methods used in the guidelines to establish the quality of the evidence on which the recommendations/statements were based were described. We also recorded some relevant sentences reported by the guidelines in the text or flowcharts but not clearly identified as recommendations/statements. When reported in the table, some complex recommendations/statements were split into multiple parts to facilitate comparisons between guidelines.

The Appraisal of Guidelines for Research and Evaluation (AGREE) Global Rating Scale (GRS) was used to evaluate the quality of the guidelines [14]. In particular, a score from 1 (lowest quality) to 7 (highest quality) was assigned to four items: process of development, presentation style, completeness of reporting, and clinical validity. The score for each item was determined after a discussion among the authors. The overall quality was defined based on the mean of the item scores: 1–3 = low; 4–5 = intermediate; 6–7 = high.

No artificial intelligence was used for this study. No specific statistical tests were applied.

## 3. Evidence Synthesis

### 3.1. Definition and Classification of PE

The EAU guidelines do not propose their own definition of PE, but rather report the one already formulated in the ICD’s 11th revision [13]. In this document, PE was renamed male early ejaculation and defined as follows: “Male early ejaculation is characterized by ejaculation that occurs prior to or within a very short duration of the initiation of vaginal penetration or other relevant sexual stimulation, with no or little perceived control over ejaculation. The pattern of early ejaculation has occurred episodically or persistently over a period of at least several months and is associated with clinically significant distress”. The EAU guidelines recognize the existence of a lifelong and an acquired PE. In addition, they report two “PE syndromes”: variable PE, and subjective PE. Variable PE is characterized by inconsistent and irregular early ejaculations (a normal variation in sexual performance). Subjective PE is characterized by subjective perception of rapid ejaculation during intercourse while ejaculation latency time (ELT) is in the normal range, or even longer (not a symptom or manifestation of disease). It is essential to note that the EAU guidelines do not specify specific time cut-offs for defining the various forms of PE.

The AUA/SMSNA guidelines distinguish between lifelong and acquired PE. Lifelong (or primary) PE is defined as “poor ejaculatory control, associated bother, and ejaculation within about 2 min of initiation of penetrative sex that has been present since sexual debut”. Acquired (secondary) PE is defined as “consistently poor ejaculatory control, associated bother, and ELT that is markedly reduced from prior sexual experience during penetrative sex”. For acquired PE, these guidelines propose “ELT under about 2–3 min” or “ELT reduction of 50% or more” as possible time cut-offs.

The ISSM guidelines state that PE is characterized by “(I) ejaculation that always or nearly always occurs prior to or within about 1 min of vaginal penetration from the first sexual experience (lifelong PE) OR a clinically significant reduction in ELT, often to about 3 min or less (acquired PE); (II) the inability to delay ejaculation on all or nearly all vaginal penetrations; (III) negative personal consequences, such as distress, bother, frustration, and/or the avoidance of sexual intimacy”. Moreover, these guidelines report the existence of variable and subjective PE, providing definitions similar to those of the EAU guidelines. Finally, they define anteportal ejaculation as “ejaculation prior to vaginal penetration”. This is considered to be the most severe form of PE and can cause difficulty conceiving.

Notably, all guidelines cite the DSM-5 definition of PE [11], but none use it directly:

“A. A persistent or recurrent pattern of ejaculation occurring during partnered sexual activity within approximately 1 min following vaginal penetration and before the individual wishes it; B. Present for at least 6 months and must be experienced on almost all or all (approximately 75–100%) occasions of sexual activity (in identified situational contexts or, if generalized, in all contexts); C. Clinically significant distress in the individual; D. The sexual dysfunction is not better explained by a nonsexual mental disorder or as a consequence of severe relationship distress or other significant stressors and is not attributable to the effects of a substance/medication or another medical condition”.

Both the EAU and ISSM guidelines specify that the definition of PE is limited to intravaginal sexual activity (not oral sex, anal sex, or masturbation), and that it cannot be applied to men who have sex with men (MSM).

### 3.2. Epidemiology and Pathophysiology of PE

The ISSM guidelines underline that several old studies characterize PE as the “most common male sexual dysfunction”, with a prevalence rate of 20–30% [15,16]. The EAU guidelines report that the highest prevalence rate of 31% was found by the National Health and Social Life Survey (NHSLS) in the USA [16]. However, both guidelines specify that it is unlikely that the prevalence is as high as 20–30%, based on the relatively low number of men who seek medical help for PE. These high prevalence rates may result from the lack of objective diagnostic criteria, the use of dichotomous or generic questions for the assessment of ejaculation, and the inclusion of men with variable and subjective PE.

Interestingly, both the ISSM and EAU guidelines describe the results of two surveys from Turkey [17] and China [18]. These studies found an overall prevalence of PE of 19.8% and 25.8%, respectively. After stratification, the rates of lifelong, acquired, variable, and subjective PE were 2.3% and 3.2%, 3.9% and 4.5%, 8.5% and 11.4%, and 5.1% and 6.4%, respectively. Moreover, both guidelines describe heterogeneous results of studies on the prevalence of PE in MSM [19].

The AUA/SMSNA guidelines state that the prevalence of PE reported in the literature is less than 5%, although in clinical practice it appears to be not at all rare. They suggest that the perception of rarity may arise from the frequency with which other sexual function disorders (e.g., ED) occur in men with PE, overshadowing it [20,21,22,23].

The ISSM guidelines affirm that, considering an ELT of about 1 min, the prevalence of lifelong PE is unlikely to exceed 4% in the general population. Moreover, they state that a prevalence approximately of 5% for acquired and lifelong PE in general populations is consistent with epidemiological data indicating that around 5% of the population has an ELT of less than 2 min.

The EAU and ISSM guidelines, but not the AUA/SMSNA guidelines, address the issue of the pathophysiology of PE. Both guidelines emphasize that the etiology of PE is unclear, that many biological and psychological risk factors have been proposed (albeit based on limited evidence), and that the subtype of PE should be considered when attempting to identify the underlying cause. The ISSM guidelines conclude that, to date, no biological factor has been shown to be causative in the majority of men with PE.

According to the EAU guidelines, lifelong PE could be mediated by a complex interplay of central and peripheral serotonergic, dopaminergic, oxytocinergic, endocrinological, genetic, and epigenetic factors; acquired PE could occur due to psychological problems (e.g., sexual performance anxiety, relationship problems) and/or comorbidities, including ED, prostatitis, hyperthyroidism, and poor sleep quality; variable PE should be considered to be a normal variation of sexual function; subjective PE could derive from cultural or abnormal psychological constructs. These guidelines underline that a significant proportion of men with ED also experience PE, possibly due to performance anxiety related to ED, with a risk of misdiagnosing PE instead of the underlying ED. Finally, the EAU guidelines list other reported risk factors for PE, including genetic predisposition, obesity, diabetes, metabolic syndrome, lack of physical activity, penile hypersensitivity, prostatitis, hyperthyroidism, hypoprolactinemia, hypertestosteronemia, low vitamin D and B12 levels, poor overall health status, low educational level, emotional problems and stress, depression, anxiety, traumatic sexual experiences, and ethnicity (Black men, Hispanic men, men from Islamic regions). However, they specify that age, marital, and income status do not appear to be related to the prevalence of PE [5,8].

The ISSM guidelines lists possible risk factors for PE, including anxiety, hypersensitivity of the glans, robust cortical representation of the pudendal nerve, alterations in central serotonergic neurotransmission, ED, prostatitis, chronic pelvic pain syndrome (CPPS), detoxification from prescribed medications, recreational drugs, and hyperthyroidism. In addition, they detail possible neurobiological (serotonin, dopamine, and oxytocin dysregulation), genetic (family history, polymorphisms of genes implicated in serotonergic and dopaminergic neurotransmission), hormonal (hyperthyroidism, hypoprolactinemia, hypertestosteronemia), prostatic (chronic prostatitis), and psychological (sexual abuse, attitudes toward sex internalized during childhood, negative body image, depression, performance anxiety, alexithymia, decreased intimacy with partner, partner conflict) risk factors. Finally, the ISSM guidelines also underline the association between PE and ED, specifying that patients can confuse these conditions (possible misdiagnosis) and suggesting that men with ED may experience PE due to performance anxiety or deliberate intensification of stimulation to ejaculate before losing their erection. It is important to highlight that, according to these guidelines, while neurobiological and genetic factors are primarily related to lifelong PE, other elements such as hyperthyroidism, chronic prostatitis, and ED are primarily related to acquired PE [5,10].

### 3.3. Recommendations/Statements on the Diagnosis and Therapy of PE

The EAU guidelines use a modified Grading of Recommendations, Assessment, Development, and Evaluations (GRADE) system to evaluate the level of evidence (1a, 1b, 2a, 2b, 3, 4) and the strength of recommendation (Strong, Weak) [24]. The AUA/SMSNA guidelines categorize the level of evidence as Grade A, Grade B, or Grade C, while they report the strength of recommendations as Strong, Moderate, or Conditional. When evidence is lacking, the AUA/SMSNA guidelines can provide statements as Expert Opinion or Clinical Principle [9]. In the ISSM guidelines, the level of evidence on which the recommendations are based is graded using the Oxford Centre for Evidence-Based Medicine (OCEBM)’s old system (1a, 1b, 1c, 2a, 2b, 2c, 3a, 3b, 4, 5) [25].

All guidelines recommend collecting medical and sexual history, underling the importance of investigating ELT, control of ejaculation, and related distress. The EAU and ISSM guidelines recommend recording the self-estimated intravaginal ejaculation latency time (IELT), while the AUA/SMSNA guidelines make no reference to this specific parameter. All guidelines agree on the importance of including a psychological evaluation of the patient; however, only the EAU guidelines recommend including the partner during the psychological assessment. All guidelines recommend the use of validated questionnaires for PE assessment. More specifically, the AUA/SMSNA guidelines do not specify which questionnaires to apply; the EAU guidelines suggest the Premature Ejaculation Diagnostic Tool (PEDT) [26], Arabic Index of Premature Ejaculation (AIPE) [27], and Premature Ejaculation Profile (PEP) [28] (also citing the Index of Premature Ejaculation [IPE] [29] and Male Sexual Health Questionnaire—Ejaculatory Dysfunction [MSHQ-EjD] [30]), while the ISSM guidelines indicate PEP and IPE (also citing PEDT, AIPE, and Chinese Index of Premature Ejaculation [CIPE] [31]). All guidelines recommend including a physical examination in the assessment of patients; however, the ISSM guidelines specify that this is highly advisable but not mandatory for lifelong PE, while it is indispensable for acquired PE. The EAU guidelines do not recommend performing routine laboratory or physiological tests (they should only be directed by specific findings from history or physical examination). The AUA/SMSNA guidelines state that additional tests may be utilized for acquired PE, while they do not recommend them for lifelong PE. The ISSM guidelines make no reference to any additional tests.

Recommendations of guidelines for the diagnosis of PE are detailed in Table 1.

The EAU guidelines recommend treating ED and prostatitis before PE. The AUA/SMSNA and ISSM guidelines simply suggest treating comorbid ED. The EAU guidelines highlight the possibility of using phosphodiesterase 5 inhibitors (PDE5Is), alone or in combination with other therapies, in patients without ED, while the ISSM guidelines state that there is not yet enough evidence to recommend PDE5Is in this subgroup. The AUA/SMSNA has no specific statements regarding PDE5Is.

The EAU guidelines recommend on-demand dapoxetine or the lidocaine/prilocaine spray as first-line treatments (in particular for lifelong PE), daily selective serotonin re-uptake Inhibitors (SSRIs) or daily/on-demand clomipramine as second-line treatments, and on-demand tramadol as a third-line treatment. The AUA/SMSNA guidelines recommend on-demand dapoxetine, topical penile anesthetics, daily SSRIs, or on-demand clomipramine as first-line treatments, followed by on-demand tramadol in men who fail first-line pharmacotherapy. The ISSM guidelines highlight the robust evidence supporting the use of on-demand dapoxetine, on-demand label topical anesthetics, daily/on-demand SSRIs, and daily/on-demand clomipramine, while they suggest using tramadol when other therapies have failed. Both the EAU and ISSM guidelines propose cautious use of tramadol due to possible adverse effects. The AUA/SMSNA guidelines consider the possibility of treating patients who have failed the first line of treatment with α1-adrenoreceptor antagonists (an alternative to tramadol), while both the EAU and ISSM guidelines do not provide any recommendations on their use.

Hyaluronic acid injections in the glans to reduce sensitivity are considered by the EAU guidelines as a therapeutic option to be used with caution, while the AUA/SMSNA consider them experimental and usable only in the context of clinical trials, and the ISSM guidelines recommend against using this treatment.

Only the AUA/SMSNA guidelines clearly state that ELT is not affected by circumcision status (indirectly discouraging this procedure as a therapy for PE). No guidelines cite frenulectomy as a possible treatment for PE. Dorsal neurectomy involves the selective cutting of the dorsal nerves of the penis to reduce its sensitivity. This surgical procedure is not recommended by the EAU and ISSM guidelines, while it is considered experimental by the AUA/SMSNA guidelines.

All guidelines suggest that psychological/behavioral therapies can be used in the treatment of PE. The EAU and AUA/SMSNA guidelines specify that psychological/behavioral therapies in combination with pharmacological treatment can improve the outcomes of patients compared to medical therapy alone. The ISSM guidelines highlight the important role of education and counselling in the management of PE. The EAU guidelines state that behavioral techniques may be beneficial in treating variable and subjective PE; the ISSM guidelines affirm that men with variable PE should be educated and reassured, while men with subjective PE may require psychotherapy.

All guidelines underline the possible negative impact of PE on the sexual satisfaction and QoL of partners, suggesting involving the partner in the management of the patient. However, the EAU guidelines consider the partner only in the section dedicated to psychotherapy, while the ISSM guidelines underline that such involvement is not mandatory.

**Figure 1:**
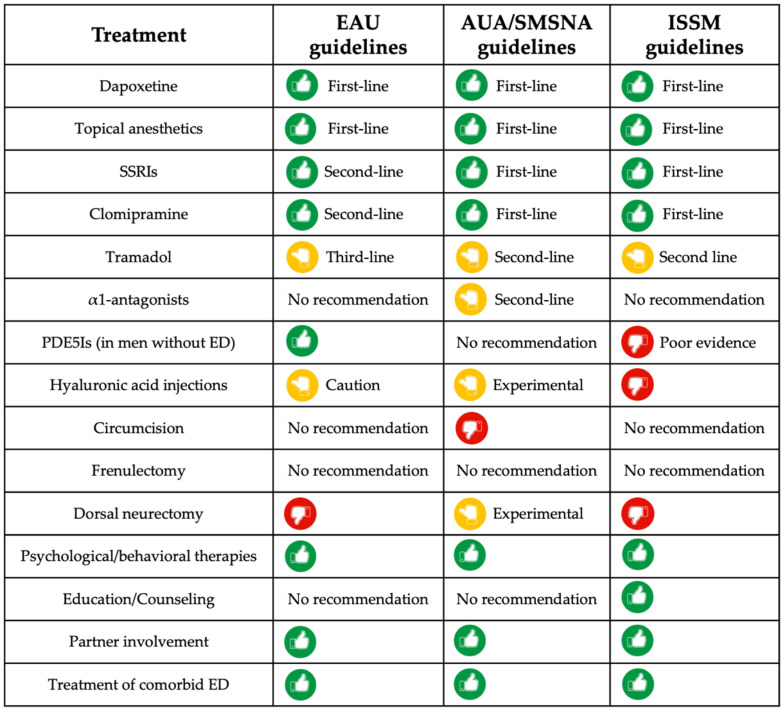
Comparison of treatments recommended by guidelines on PE.

Recommendations of guidelines for the treatment of PE are listed in Supplementary Table S1. A comparison of treatments recommended by the guidelines on PE is shown in Figure 1.

The comparison refers to the EAU guidelines 2024, AUA/SMSNA guidelines 2022, and ISSM guidelines 2014.

AUA/SMSNA: American Urological Association/Sexual Medicine Society of North America; EAU: European Association of Urology; ED: erectile dysfunction; ISSM: International Society of Sexual Medicine; PDE5I: phosphodiesterase 5 inhibitor; PE: premature ejaculation; SSRI: selective serotonin re-uptake inhibitor.

### 3.4. Quality of Guidelines on PE

No significant differences were found among the guidelines for the “process of development” and “clinical validity” items of AGREE GRS, which were good in all cases. “Presentation style” and “completeness of reporting” were excellent for the ISSM guidelines, while the AUA/SMSNA guidelines appeared to be less well structured and lacking in some topics (epidemiology was only mentioned, and pathophysiology was missing). The overall quality was rated high for the ISSM and EAU guidelines, while it was considered intermediate for the AUA/SMSNA guidelines (mean scores: 6.5, 6.0, and 5.3 points, respectively). However, in the context of quality assessment, it is essential to underline that the EAU guidelines are the most recent (2024), while the ISSM guidelines are the least updated (2014).

**Figure 2:**
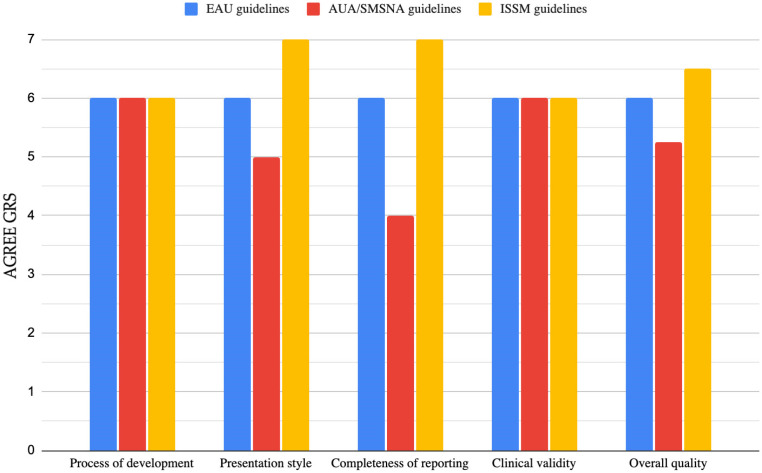
Quality of guidelines on PE according to AGREE GRS [14].

The AGREE GRS for the evaluated guidelines is shown in Figure 2.

The evaluation refers to the EAU guidelines 2024, AUA/SMSNA guidelines 2022, and ISSM guidelines 2014.

AGREE: Appraisal of Guidelines for Research and Evaluation; AUA/SMSNA: American Urological Association/Sexual Medicine Society of North America; EAU: European Association of Urology; GRS: Global Rating Scale; ISSM: International Society of Sexual Medicine; PE: premature ejaculation.

### 3.5. Strengths, Limitations, and Future Perspectives

This is the most up-to-date review of the PE guidelines. It provides an in-depth comparison of the main international guidelines in force, assessing their quality, highlighting knowledge gaps on the topic, and reporting differences regarding the recommended diagnostic and therapeutic paths. Consequently, it could address future research efforts, promote the improvement of guidelines, and ultimately favor the standardization of the management of PE patients.

However, our paper should be read and interpreted considering its limitations. In particular, the selection of the guidelines, the choice of evidence to report, and the assessment of the guidelines’ quality were arbitrary by the authors (although based on their experience on the topic and resulting from appropriate discussions). Furthermore, it should be taken into account that the guidelines analyzed were published in different years; therefore, some of the differences detected could be based on the evidence available at the time of release; in this regard, it should be remembered that the ISSM guidelines are the oldest, while the EAU guidelines are the most up-to-date. Finally, the purpose of this article was to examine similarities and differences between current PE guidelines, not to analyze the available evidence to judge which guideline is most correct or propose new recommendations—tasks that fall to scientific societies.

The elaboration of a unified definition of PE with clear cut-off criteria is essential for advancing research and clinical practice. Efforts should be directed towards a correct classification and evaluation of PE following non-intravaginal sexual activities and in MSM. Large epidemiological studies using a universally accepted definition and validated assessment tools are needed to better understand the prevalence of PE in general populations and subgroups. Further research into the neurobiological and psychological aspects of PE is crucial to elucidate the underlying mechanisms and improve treatment approaches. Additionally, more studies are required to better explore the correlations between PE and conditions such as prostatitis, hyperthyroidism, and ED to strengthen the evidence already available. Finally, the standardization of diagnostic and therapeutic pathways would guarantee consistent and optimal management of PE worldwide, improving patient outcomes. These algorithms should be based on high levels of evidence (therefore, new high-quality studies are needed) and should differ depending on the subtype of PE.

## 4. Conclusions

Different international guidelines on PE are currently in force. They have several similarities but also many differences. The latter appear particularly evident when analyzing the definitions of PE and the recommendations on the management of the patient with PE. It is essential to start from this comparison to understand knowledge gaps, promote studies to improve the evidence on the topic, and ultimately encourage the formulation of a shared definition and the development of standardized diagnostic–therapeutic algorithms by the main international scientific societies.

## Figures and Tables

**Table 1:** Recommendations/statements for diagnosis of PE.

EAU Guidelines	AUA/SMSNA Guidelines	ISSM Guidelines
***Medical and sexual history***		
Perform the diagnosis and classification of PE based on medical and sexual history, which should include assessment of IELT (self-estimated), perceived control, distress, and interpersonal difficulty due to the ejaculatory dysfunction. *Strong*	Clinicians should assess medical, relationship, and sexual history to make the diagnosis of PE. *Clinical Principle* Query on the absence or loss of ejaculatory control, personal or interpersonal bother, and short ELT is essential. *NA*	It is recommended take a medical history. *LOE 5* It is recommended that clinicians utilize the screening questions: - Recommended questions for diagnosis: What is the time between penetration and ejaculation (cumming)? Can you delay ejaculation? Do you feel bothered, annoyed, and/or frustrated by your PE? - Optional questions to differentiate lifelong and acquired PE: When did you first experience PE? Have you experienced PE since your first sexual experience on every/almost every attempt and with every partner? *NA* It is recommended that self-estimation by the patient and partner of ejaculatory latency be accepted as the method for determining IELT in clinical practice. *LOE 2b* There was inadequate evidence to recommend screening or case finding for PE, either in a general population or in any subpopulation. However, it is recommended that men with ED be screened for PE. *NA*
***Psychological evaluation***		
Assess psychosexual history and psychosexual development. *Strong* Assess anxiety and interpersonal anxiety; focus on control issues. *Strong* Include the partner if available (in the psychological assessment); check for the impact of PE on the partner. *Strong*	A psychological health assessment should also be obtained. *NA*	It is recommended to take a psychosocial history. *LOE 5*
***Physical examination***		
Include physical examination in the initial assessment of PE to identify anatomical abnormalities that may be associated with PE or other sexual dysfunctions, particularly ED. *Strong*	Clinicians should perform a focused physical exam to make the diagnosis of PE. *Clinical Principle*	For lifelong PE, a physical examination is highly advisable but not mandatory and should be conducted in most if not all patients. *NA* For acquired PE, a targeted physical examination is mandatory to assess for associated/causal diseases such as ED, thyroid dysfunction, or prostatitis. *NA*
***Questionnaires***		
Use patient-reported outcomes in daily clinical practice. *Weak* Several questionnaires can be used for the diagnosis of PE (PEDT, AIPE) and for assessing the therapeutic outcomes of PE interventions (PEP). *LOE 2b*	Clinicians may use validated instruments to assist in the diagnosis of PE. *Conditional Recommendation; LOE: Grade C*	Depending on the specific need, the PEP or IPE continue to be the preferred questionnaire measures for assessing lifelong or acquired subtypes of PE, particularly when monitoring responsiveness to treatment. *LOE 2b*
***Other tests***		
Do not perform routine laboratory or physiological tests. They should only be directed by specific findings from history or physical examination. *Strong*	Clinicians should not use additional testing for the evaluation of a patient with lifelong PE. *Conditional Recommendation; LOE: Grade C* Clinicians may utilize additional testing as clinically indicated for the evaluation of the patient with acquired PE. *Conditional Recommendation; LOE: Grade C*	No statements.

The recommendations reported refer to the EAU guidelines 2024, AUA/SMSNA guidelines 2022, and ISSM guidelines 2014. AIPE: Arabic Index of Premature Ejaculation; AUA/SMSNA: American Urological Association/Sexual Medicine Society of North America; EAU: European Association of Urology; ED: erectile dysfunction; ELT: ejaculation latency time; IELT: intravaginal ejaculation latency time; IPE: Index of Premature Ejaculation; ISSM: International Society of Sexual Medicine; LOE: level of evidence; NA: not available; PE: premature ejaculation; PEDT: Premature Ejaculation Diagnostic Tool; PEP: Premature Ejaculation Profile.

## Data Availability

This is a review; therefore, all data were sourced from the articles cited.

## Supplementary Materials

The following supporting information can be downloaded at: https://www.mdpi.com/article/10.3390/diagnostics14161819/s1, Table S1: Recommendations/Statements for treatment of PE.
