# Supplementary material for: Comparison of Current International Guidelines on Premature Ejaculation: 2024 Update
Source: Diagnostics (Basel). 2024 Aug 21;14(16):1819. doi: 10.3390/diagnostics14161819 (PMC11353472; doi:10.3390/diagnostics14161819)
Supplement: Supplementary file 1 [file diagnostics-14-01819-s001.zip › diagnostics-3119737-supplementary.pdf]

Supplementary Table S1. Recommendations/Statements for treatment of PE

EAU guidelines	AUA/SMSNA guidelines	ISSM guidelines
Treat ED, other sexual dysfunction or genitourinary infection (e.g.,prostatitis) first. *Strong*<br><br>Use either dapoxetine or the lidocaine/prilocaine spray as first-line treatments for lifelong PE. *Strong*<br><br>Use off-label oral treatment with daily SSRIs or daily/on-demand clomipramine as a viable alternative for second-line treatments. *Strong*<br><br>Use off-label tramadol with caution as a viable on-demand third-line treatment alternative to on-demand/daily antidepressants (SSRIs or clomipramine). *Strong*<br><br>Use PDE5Is alone or in combination with other therapies in patients with PE (without ED). *Strong*<br><br>Hyaluronic acid injections are effective in decreasing penile sensitivity. *LOE 2b*<br>Use hyaluronic acid injection with caution as a treatment option for PE compared to other more established treatment modalities. *Weak*<br><br>Do not perform dorsal neurectomy because more safety data are warranted. *Weak*<br><br>Decide on referral to (sexual)psychotherapy; include partner actively. *Strong*<br><br>The combination of psychosexual approaches and pharmacological treatments yields superior outcomes compared to pharmacological interventions alone. *LOE 3*<br>Use psychological/behavioral therapies in combination with pharmacological treatment in the management of acquired PE. *Weak*<br><br>Various behavioral techniques may be beneficial in treating variable and subjective PE. *NA*	Clinicians should treat comorbid ED in patients with PE according to the AUA Guidelines on ED. *Expert Opinion*<br><br>Clinicians should recommend daily SSRIs; on demand clomipramine or dapoxetine (where available); and topical penile anesthetics as first-line agents of choice in treatment of PE. *Strong Recommendation; LOE: Grade B*<br><br>Clinicians may consider on-demand dosing of tramadol for treatment of PE in men who have failed first-line therapy pharmacotherapy. *Conditional Recommendation; LOE: Grade C*<br><br>Clinicians may consider treating men with PE who have failed firstline therapy with a1-adrenoreceptor antagonists. *Expert Opinion*<br><br>Clinicians should advise patients that there is insufficient evidence to support the use of alternative therapies in the treatment of PE. *Expert Opinion*<br><br>Clinicians should inform patients that surgical management (including injection of bulking agents) of PE should be considered experimental and only be used in the context of an ethical board-approved clinical trial. *Expert Opinion*<br><br>Clinicians should advise patients that ejaculatory latency is not affected by circumcision status. *Conditional Recommendation; LOE: Grade C*<br><br>Clinicians should consider referring men diagnosed with PE to a mental health professional with expertise in sexual health. *Moderate Recommendation; LOE: Grade C*<br><br>Clinicians should advise men with PE that combining behavioral and pharmacological approaches may be more effective than either modality alone. Moderate Recommendation; *LOE: Grade B*<br><br>Involvement of sexual partner(s) in decision making, when possible, may allow for optimization of outcomes. *NA*	There is reliable evidence to support the treatment of PE and comorbid ED with ED pharmacotherapy in combination with PE pharmacotherapy. *LOE 3c*<br><br>There is robust evidence to support the efficacy and safety of on-demand dosing of dapoxetine for the treatment of lifelong and acquired PE. *LOE 1a*<br><br>There is robust evidence to support the efficacy and safety of off-label daily dosing of the SSRIs paroxetine, sertraline, citalopram, fluoxetine, and the serotonergic tricyclic clomipramine, and off-label on-demand dosing of clomipramine, paroxetine, and sertraline for the treatment of of lifelong and acquired PE. *LOE 1a*<br><br>There is good evidence to support the efficacy and safety of off-label on-demand label topical anesthetics in the treatment of longlife PE. *LOE 1a*<br><br>Tramadol may be an effective option for the treatment of PE. However, it may be considered when other therapies have failed because of the risk of addiction and side effects. It should not be combined with an SSRI because of the risk of serotonin syndrome, a potentially fatal outcome. Further well-controlled studies are required to assess the efficacy and safety of tramadol in the treatment of PE patients. *LOE 2*<br><br>There is some evidence to support the efficacy and safety of off-label on-demand or daily dosing of PDE5Is in the treatment of longlife PE in men with normal erectile function. *LOE 4d*<br>Treatment of lifelong PE with PDE5Is in men with normal erectile function is not recommended, and further evidence-based research is encouraged to further understand conflicting data. *NA*<br><br>In one human study, an oxytocin antagonist failed to clinically or statistically improve IELT. Further human studies are necessary. *LOE 4*<br>Intracavernosal injection of vasoactive drugs is not recommended for the treatment of PE. *LOE 4*<br>There are limited positive data regarding the effectiveness of acupuncture therapy. *LOE 3b*<br><br>Neuromodulation of the dorsal penile nerve is an invasive and irreversible procedure, which is associated with an increase in the IELT. However, safety of this treatment modality needs to be determined before this procedure can be recommended for treating PE patients. *LOE 4*<br>Selective dorsal nerve neurotomy or glans penis augmentation using hyaluronic acid gel may be associated with permanent loss of sexual function and is not recommended in the management of PE. *NA*

		There is some evidence to support the efficacy of psychological and behavioral interventions in the treatment of PE. *LOE 2b*<br><br>Combining a medical and psychological approach may be especially useful in men with acquired PE where there is a clear psychosocial precipitant or lifelong cases where the individual or couple's issues interfere in the medical treatment and success of therapy. Similarly, in men with PE and comorbid ED, combination therapy may also be helpful to manage the psychosocial aspects of these sexual dysfunctions. *LOE 2a*<br><br>Educational or coaching strategies are designed to give the man the confidence to try the medical intervention, reduce performance anxiety, and modify his maladaptive sexual scripts. *LOE 5*<br><br>Education on the nature of PE, helping men improve ejaculatory control with behavioral exercises, addressing restricted/narrow sexual behavioral patterns, and resolving interpersonal issues are likely to be of significant help to men with acquired PE. Once the man's self-confidence and sense of control have improved, it may then be possible to reduce or discontinue the medical intervention. *LOE 5*<br><br>If the PE has resulted in psychological and relationship concerns, graded levels of patient and couple counseling, guidance, and/or relationship therapy may be a useful adjunct to the medical intervention. *LOE 1a*<br><br>Men with variable PE should be educated and reassured. Men with subjective PE may require a referral for psychotherapy. More research is necessary to better define the efficacy of reassurance, education, and psychotherapy with these provisional subtypes. *NA*<br><br>Inclusion of the partner in the treatment process is an important but not a mandatory ingredient for treatment success. *NA*

The recommendations reported refer to EAU guidelines 2024, AUA/SMSNA guidelines 2022, ISSM guidelines 2014.
AUA/SMSNA: American Urological Association/Sexual Medicine Society of North America; EAU: European Association of Urology; ED: Erectile Dysfunction; IELT: Intravaginal Ejaculation Latency Time; ISSM: International Society of Sexual Medicine; LOE: Level Of Evidence; NA: Not Available; PDE5I: Phosphodiesterase 5 Inhibitor; PE: Premature Ejaculation; SSRI: Selective Serotonin Re-uptake Inhibitor.
